# Revitalizing dendritic lithium with atomic modulator-decorated suspension electrolyte for durable lithium metal batteries

**DOI:** 10.1126/sciadv.aef9111

**Published:** 2026-05-22

**Authors:** Jian Wang, Bingbing Tang, Jian Wu, Jing Zhang, Zhenjiang Cao, Hao Li, Yidong Miao, Huihua Li, Fanglin Wu, Fangqi Liu, Dong Wang, Yongzheng Zhang, Qingbo Xiao, Hongzhen Lin, Maximilian Fichtner

**Affiliations:** ^1^ Helmholtz Institute Ulm (HIU), Ulm D89081, Germany.; ^2^ Institute of Nanotechnology (INT), Karlsruhe Institute of Technology (KIT), Hermann-von-Helmholtz-Platz 1, Karlsruhe D76344, Germany.; ^3^ *i*-Lab and CAS Key Laboratory of Nanophotonic Materials and Devices, Suzhou Institute of Nano-tech and Nano-bionics, Chinese Academy of Sciences, Suzhou 215123, China.; ^4^ College of Advanced Interdisciplinary Studies, National University of Defense Technology, Changsha 410073, China.; ^5^ School of Materials Science and Engineering, Xi’an University of Technology, Xi’an 710048, China.; ^6^ School of Chemistry, Engineering Research Center of Energy Storage Materials and Devices, Xi’an Jiaotong University, Xi’an 710049, China.; ^7^ Key Laboratory of Automobile Materials of MOE, School of Materials Science and Engineering, Jilin University, Changchun, 130013, China.; ^8^ School of Textile and Clothing, Nantong University, Nantong 226019, China.

## Abstract

Lithium metal anodes suffer from dendrite growth, unstable solid electrolyte interphase, and “dead Li” owing to high barriers and inhomogeneous Li^+^ desolvation/diffusion kinetics. Here, we present the suspension electrolyte endowed with atom-level catalytic inorganic particles of single atomic cobalt on defect-rich ZnO_1−_*x*__ nanoparticles (SACo@ZO) in a carbonate-based electrolyte, enhancing desolvation/diffusion kinetics and revitalizing dendritic Li. As systematically investigated by in situ electrochemical sum frequency generation (SFG) spectroscopy together with theoretical simulations, the SACo@ZO-assisted suspension electrolyte decreases the potential threshold down to 20 millivolts for driving interfacial desolvation rapidly, providing uniform solvation-free Li^+^/Li^0^ flux and capability in revitalizing dendritic Li. Consequently, we achieve a smooth but dense Li plating behavior under room or low-temperature surroundings, lasting for a long life span of 1600 hours. Meanwhile, the practical Li-LiFePO_4_ cell with SACo@ZO reserves the capacity retention of ~100% at 0.5 C and survives for 1000 cycles under 0°C, demonstrating the feasibility of atomically catalytic suspension electrolyte for high-performance dendrite-free Li metal batteries.

## Teaser

Suspension electrolyte with atomic catalytic inorganic particles accelerates interfacial desolvation and revitalizes dendritic Li.

## INTRODUCTION

**Fig. 1.**
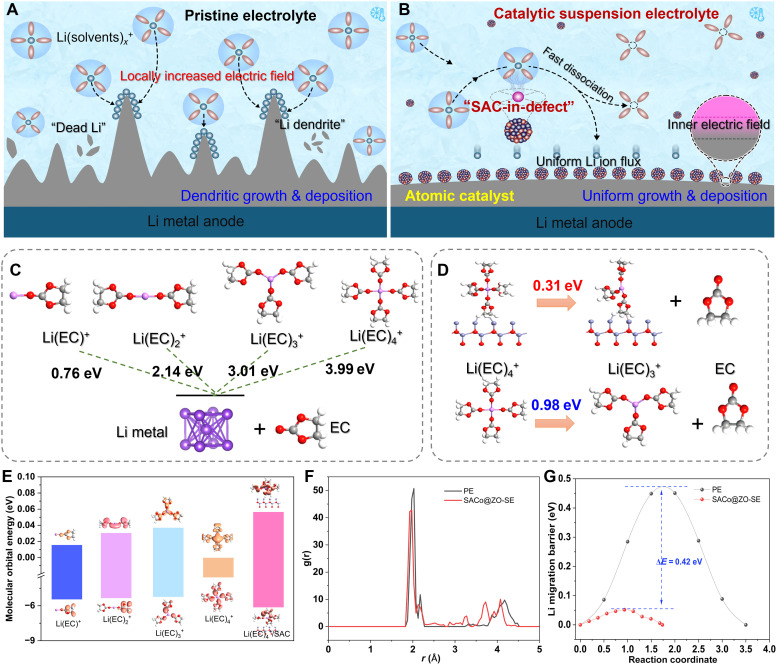
Schematic illustration and theoretical simulation with atomic modulators on electrode/electrolyte interface. The illustration of the solvation shell and corresponding Li atom plating behaviors under (**A**) pristine commercial electrolyte and (**B**) SAC-decorated SE. (**C**) The reduction barrier evolution of the Li(EC)_4_^+^ species to Li metal and EC molecular. (**D**) Comparison of desolvation barrier from Li(EC)_4_^+^ to Li(EC)_3_^+^ and EC with/without SAC-decorated suspension particles. (**E**) The molecular orbital energy of each species in the electrolyte with/without SAC-decorated suspension particles. (**F**) The coordination environment and distribution of Li^+^ solvation shell in the two electrolytes. (**G**) The comparison of Li migration barrier in the electrolyte with/without SAC catalyst.

Metallic lithium anode is a promising alternative in next-generation battery systems because of high theoretical capacity (3860 mA hour g^−1^) and the lowest reduction potential (−3.04 V versus standard hydrogen electrode) (*1*–*3*). However, Li metal anode often suffers from the sluggish diffusion kinetics of Li ion/atom (*4*–*6*), leading to inhomogeneous Li^+^ distribution and successive formation of dendritic Li (*7*). As known, the dendritic Li formation can be attributed to partial Li atom aggregation in the local region with limited lateral diffusion kinetics. Meanwhile, the Li^+^ solvation chemistry is also responsible for the Li plating/stripping performance (Fig. 1A) (*8*). The primary Li^+^ solvation sheath surrounding exerts a field-screening effect to the central charge, which facilities the migration and diffusion of solvated Li^+^ but often decreases its mobility due to the large steric hindrance (*9*, *10*). The Li(solvents)*_x_*^+^-(anions)^−^ solvation clusters engineered by electrolyte chemistry have the close relationship even decisive role in the formation of solid electrolyte interphase (SEI) (*11*). This allows constructing a more resilient SEI with rich inorganic compounds like LiF for smoother and more uniform plating. Accordingly, the freshly generated Li^0^ atoms will unevenly nucleate with high barriers, leading to fractal growth of Li dendrites (*12*, *13*). The polarized solvation sheath is electron-insufficient, and regulating the Li^+^ solvation chemistry via salts or solvents has become the essential way to make lithium metal batteries (LMBs) successful. However, it remains challenging to revitalize dendritic Li with simple methods, which leads to short circuit and severe safety (*8*, *14*, *15*).

Strategies for optimizing SEI functionality and enhancing ion transport primarily encompass two approaches: (i) interphase engineering with extra fast ion diffusion property for regulating the kinetics and (ii) electrolyte engineering with designed solvation structures of Li(solvents)*_x_*^+^ (*16*–*20*). Although these interphase and host strategies are somehow effective in facilitating the ion transport across the interphase along with SEI reconstruction, tedious extra processing steps such as extra coating and drying are inevitable. Alternatively, electrolyte engineering from solvent optimizations to electrolyte selections to modulate ion-dipole and dipole-dipole interactions has been pursuit due to its potential large-scale production feasibility and as-expected SEI ingredient and morphology (*21*, *22*). However, the high-concentration electrolytes mostly aim at forming inorganic-rich robust SEI by increasing the content of anions in the local solvation structure (*23*, *24*). The diluent solvent strategy regulates the solvation shell of Li(solvents)*_x_*^+^ (*25*), but it inevitably results in low conductivity and sluggish ion transport (*26*). Reduced to low-temperature surroundings, the energy barriers of desolvation to generate free Li^+^ at the interface become even higher (*15*, *27*). In principal, besides the modulation of the thermodynamically equilibrated state of the solvation structure, a more important issue is to increase the solvation/desolvation rate constants, so that the solvated and desolvated state can be readily transformed between each other whenever required.

Despite SEI, to reactivate dendritic Li and “dead Li,” the main method is that introducing the redox mediators such as I^−^/I_3_^−^ into the electrolyte or adopting discharged state calendar protocol (*5*, *28*, *29*). Unfortunately, the revitalizing efficiency is still far from practical demand. Alternatively, insoluble inorganic electrolyte additives as suspension particles have emerged to change the solvation environments in the suspension electrolyte (SE), which is proved to effectively regulate the SEI evolution and Li^+^ behaviors (*30*–*33*). However, the participation of Li_2_O or Li_3_N nanoparticles in the solvation shell structure only works efficiently in the fresh electrodes, and it failed in revitalizing dendritic Li and delocalizing atomic aggregation after the long-term cycles (*34*). Instead of perfect nanoparticles, single-atom catalysts (SACs) behave the best in decreasing barriers thanks to the merit of highest activity (*35*–*40*). Our previous works had pioneered the concept of “SAC-in-Defect” and demonstrated that SAC-decorated interphase layers are capable of accelerating interfacial desolvation and diffusion as well (*41*–*43*). However, it remains to respond how SAC-decorated SE behave and affect the Li plating behaviors against Li dendrite.

Here, the SE used with atomic modulators has been initially proposed to revitalize dendritic Li, where the catalytic nanoparticles serve as Li^+^ solvation modulators for further uniformizing spatial Li ion/atom distribution to revitalize dendritic Li, as illustrated in Fig. 1B. As a proof of concept, the single atomic cobalt is located on the defect-rich ZnO_1−*x*_ (SACo@ZO), well-dispersing in the carbonate-based pristine electrolyte (PE). As fully revealed by in situ sum frequency generation (SFG) spectroscopy and theoretical simulations, the interfacial desolvation potential threshold is decreased from 120 to 20 mV with SACo@ZO, exhibiting accelerated desolvation and diffusion kinetics. The postmortem dendritic Li electrode is well revitalized under the SACo@ZO-based suspension electrolyte (SACo@ZO-SE) by delocalizing the accumulated Li atom aggregations with reduced lateral diffusion barriers, as confirmed by theoretical simulations and electronic microscopy. Consequently, a smooth but dense Li plating behavior is observed under SACo@ZO-SE at room or low temperatures, and the SACo@ZO-SE enables a high areal loading LiFePO_4_ pouch cell to deliver an ultrahigh capacity of 135 mA hour g^−1^ after 120 cycles at 0.5 C and lasts for 1000 cycles under 0°C, demonstrating the feasibility for revitalizing dendritic Li in practical LMBs.

## RESULTS

### Theoretical simulation with atomic modulators on electrode/electrolyte interface

In comparison to other sites with higher energy barriers, the lowest energy is achieved when the Co atom is used on the defect-rich sites of ZnO_1−*x*_ (fig. S1) (*44*), indicating the electron delocalization and chemical stability for the “SAC-in-Defects” structure. To assess the catalytic capability of SACo@ZO suspension particles in dissociating Li(solvents)*_x_*^+^ such as Li(ethylene carbonate)_4_^+^ [Li(EC)_4_^+^], the gradual reduction energy was calculated by varying the coordination numbers in the Li(solvents)*_x_*^+^ clusters (Fig. 1C) (*42*, *45*). Obviously, the higher barriers need to be overcome to reach free Li^+^ from Li(EC)*_x_*^+^, ranging from 0.76 to 3.99 eV. Representatively, the pristine Li(EC)_4_^+^ cluster costs the energy of 0.98 eV to drive the dissociation process into Li(EC)_3_^+^ and isolated free EC molecular (Fig. 1D), while this desolvation process only needs 0.31 eV in the SACo@ZO-based SE system, only one-third barrier of the PE. As exhibited in the highest occupied molecular orbital and the lowest unoccupied molecular orbital (Fig. 1E), the SACo@ZO-SE also broadens the redox capability of the electrolyte, which is beneficial for achieving high-voltage LMBs (*8*). The radial distribution function (RDF) displays that the Li-O peak located at 2.03 Å assigned to the interactions between Li^+^ and O-related solvents in the PE (Fig. 1F), while this peak intensity is shifted to 1.93 Å within the SACo@ZO-SE, showcasing the decreased coordination number from 4 to 3.9 (*46*). The Li atom on the Li surface is hard to diffuse because of the high barrier (0.47 eV) (Fig. 1G and fig. S2). In contrast, the Li atom is easy to diffuse around the SACo localized sites, exhibiting the barrier of 0.056 eV, about eight times lower than that on metallic Li surface, confirming the sharp contributions of SACo@ZO in accelerating Li diffusion (*12*).

### Interface evolution characterized by in situ spectroscopies

**Fig. 2.**
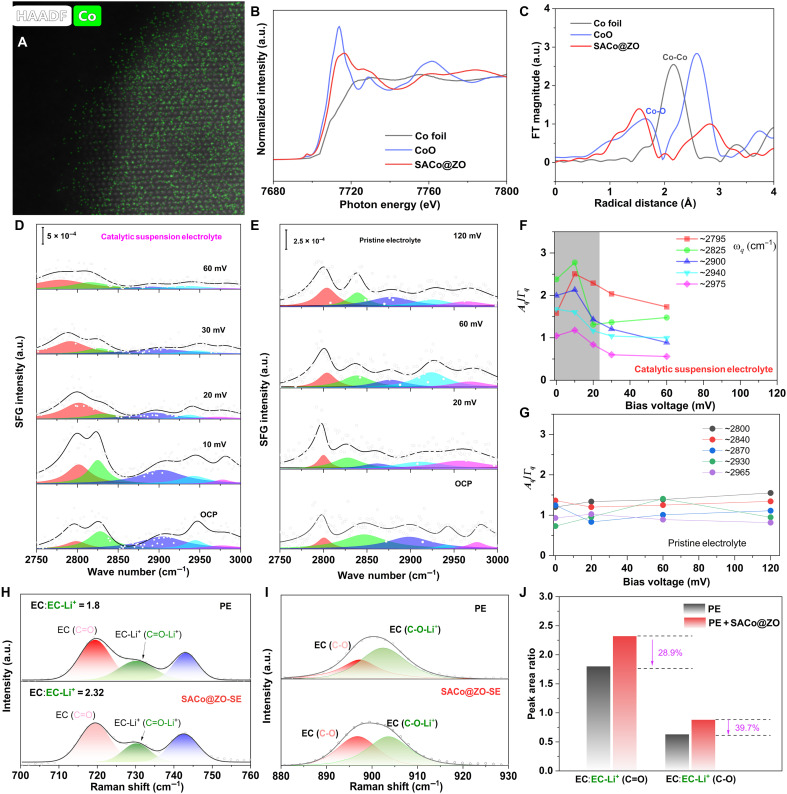
Interface evolution characterized by in situ spectroscopies. The presence of atomic Co in defect-rich ZO as probed by (**A**) HAADF-STEM with atom mapping; (**B**) the normalized XAS spectra of Co K-edge of the SACo@ZO and references; (**C**) the corresponding Fourier transformed (FT) curves of the Co K-edge EXAFS spectra of SACo@ZO and references; the SFG spectra evolution probed at the (**D**) Li/SACo@ZO-SE and (**E**) Li/PE interface under different bias voltages; the intensity summary of the C─H bond vibration along time at the (**F**) Li/SACo@ZO-SE and (**G**) Li/PE interface; (**H** and **I**) Raman spectra of the two electrolyte/Li interface; (**J**) the summary of the Raman peak area ratio. a.u., arbitrary units.

Figure S3 illustrates the fabrication process of SACo@ZO catalytic nanoparticle. The scanning electron microscopy (SEM) depicts that the particle size is around 200 nm, and the elemental Co coincides with Zn and O (fig. S4). High-resolution transmission electron microscopy in fig. S5 shows that the crystal fringe is extended to 0.234 nm. As displayed in the Fig. 2A, the bright dots observed in the high-angle atom-level dark field scanning transmission electron microscopy (HAADF-STEM) image are assigned to Co atoms as confirmed by atomic mappings (fig. S6) (*36*, *40*, *47*). The detailed chemical states and surroundings of SACo@ZO were further determined by x-ray absorption spectroscopy (XAS) (Fig. 2B). In comparison with Co foil, the SACo@ZO exhibits a shifted photon energy position with proper electron delocalization. As illustrated in the Fourier transformed (FT) curves of the extended x-ray absorption fine structure (EXAFS), the SACo@ZO exhibits a main peak at about 1.53 Å (Fig. 2C), which might be assigned to the formation of Co─O bond. Meanwhile, no Co─Co bond is observed as homogeneously isolated distribution of atomic Co in the ZnO_1−*x*_, which is consistent with the x-ray diffraction (XRD) result (fig. S7). In the high-resolution spectra (fig. S8, A to C), peaks at 1021.8 and 1044.9 eV assignable to Zn 2p_3/2_ and Zn 2p_1/2_, respectively, shifted to higher energy, and the oxygen defect peak ratio is higher than pristine ZnO (0.8 versus 0.64) (*48*). Meanwhile, the peaks at 781 and 786.4 eV are featured as Co^2+^ and satellite, respectively (fig. S8D) (*49*), which is consistent with the XAS results, indicating the electronic density redistribution by anchoring Co atom.

The dynamic solvation structure evolution of Li(solvents)*_x_*^+^ in SACo@ZO-SE has initially been investigated by the in situ SFG and Raman spectroscopy (*15*, *50*, *51*). The schematic illustration of in situ SFG is presented with the access to the Li/electrolyte interface (fig. S9). Obviously, the vibration peaks at ~2800, 2825, and ~2940 cm^−1^ assigned to the different vibration modes of C─H bond of the solvents are detected with bias voltage or at the open circuit potential (OCP), indicating the preferential adsorption of solvent molecules at the interface, which is consistent with our previous results (*16*, *52*). Slightly increasing the bias voltage, the SFG peak intensity increases accordingly, suggesting that the higher electrical field drives more Li(solvents)*_x_*^+^ species to enter the inner Helmholtz plane layer to adsorb on the Li metal surface (Fig. 2, D and E). Both SACo@ZO-SE and PE systems have the similar trends in SFG intensity of C─H bond, that is, the SFG intensity of C─H bond increases along with the increase of initial bias voltages. However, owing to the catalytic capability of SACo@ZO, the SFG peak intensity of C─H decreases with further improvements of bias voltage. As summarized in Fig. 2F, for the SACo@ZO-assisted catalytic SE system, the peak intensities of solvents in the solvation shell are significantly reduced, and most of solvents are escaping from the interfacial surface after applying 20 mV on the system, indicating that the SACo@ZO is beneficial for accelerating the Li(solvent)*_x_*^+^ dissociation to release free solvent far from the interface. However, for the PE system, the desolvation threshold is held above 60 or 120 mV (Fig. 2G), implying the higher barrier to overcome sluggish ion kinetics. The desolvation behaviors are also observed in the Raman spectroscopy, and the peaks around 718 and 905 cm^−1^ represent solvated EC-Li^+^ and free EC solvent (Fig. 2, H and I), respectively (*30*, *31*). As exhibited in Fig. 2J, the peak area ratios between free EC and Li^+^-EC are indicated as an index to observe the solvation. Compared to PE with free EC, the peak ratios of Li^+^-EC are increased by ~29 and ~40%, respectively. Above all, the in situ SFG and Raman spectroscopy results have completely demonstrated that the SACo@ZO can affect the inner Helmholtz plane layer and speed up the desolvation kinetics at the interface.

### Accelerated Li diffusion kinetics with atomic modulators in SE

**Fig. 3.**
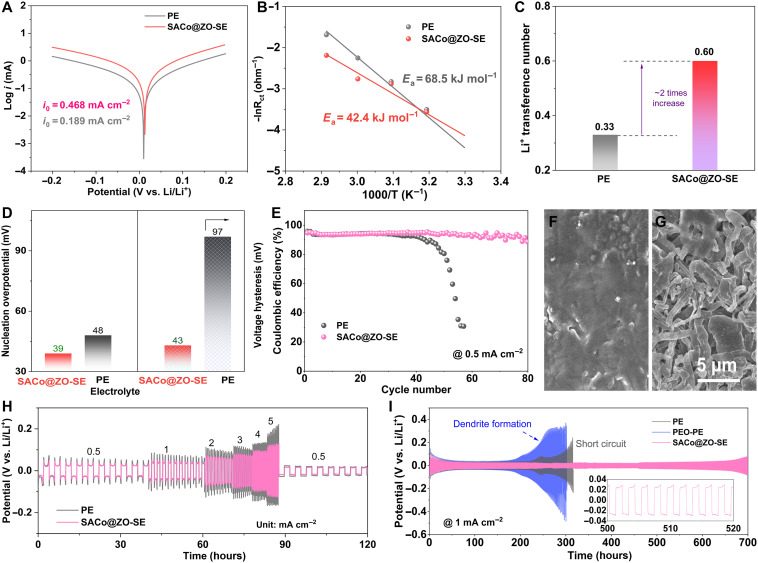
Accelerated Li diffusion kinetics with atomic modulators. (**A**) The Tafel plots, (**B**) the comparison of activation energy barriers, and (**C**) the Li ion transference number under the PE and SACo@ZO-SE electrolyte, respectively. (**D**) Comparisons of nucleation overpotential and voltage hysteresis of the Li-Cu cell used with electrolytes. (**E**) Comparison of Coulombic efficiency at 0.5 mA cm^−2^. The cycled morphology of Cu with Li plating under (**F**) SACo@ZO-SE and (**G**) PE electrolyte. The (**H**) rate performance and (**I**) life-span measurement of the Li-Li with PE, PEO-PE, or SACo@ZO-SE.

As shown in Fig. 3A, the ion diffusion kinetics are evaluated, and the larger exchange current density of 0.468 mA cm^−2^ is achieved in the SACo@ZO-SE, about three times higher than PE (0.189 mA cm^−2^). Successively, the activation energy barrier is reduced for Li^+^ desolvation under the SACo@ZO-SE (Fig. 3B and fig. S10) from 68.5 to 42.4 kJ mol^−1^. Meanwhile, the Li^+^ transference number is also enhanced from 0.33 for PE to 0.6 for SACo@ZO-SE (Fig. 3C and fig. S11). Cycled at 0.5 mA cm^−2^, the nucleation overpotential on the Cu electrode is decreased from 48 mV for PE to 39 mV for SACo@ZO-SE (Fig. 3D and fig. S12). Meanwhile, the voltage hysteresis of stripping and plating is also shortened within 43 mV, only about half of the PE one (97 mV). Further cycling, SACo@ZO-SE stabilizes that the Coulombic efficiency over the cell with PE had the limited life of 40 cycles (Fig. 3E), indicating the inhibition of dendrite formation. As inspected by SEM (Fig. 3, F to G, and fig. S13), the dendritic morphology is exhibited in the PE, while it keeps smooth plating surface under SACo@ZO-SE, indicating the ion/atom mobility at the interface.

The enhanced kinetics with SACo@ZO-SE is also shown in the smaller charge transfer resistance than PE (62 versus 139 Ω) (fig. S14). Gradually increasing current densities from 0.5 to 5 mA cm^−2^ (Fig. 3H), the overpotentials increase accordingly. However, the SACo@ZO-SE only maintains 119 mV even at 5 mA cm^−2^. Successively, the cell with SACo@ZO-SE exhibits outstanding life span over 700 hours, and the overpotential stabilizes at 24 mV under 1 mA cm^−2^ (Fig. 3I). In sharp contrast, the cell with PE only survives for about 200 hours and then encounters potential fluctuation, suggesting dendritic Li growth. Enhanced to 2 mA cm^−2^, the SACo@ZO-SE enables to last for 400 hours with the overpotential of 30 mV, whereas cells with polyethylene oxide–decorated pristine electrolyte (PEO-PE) and PE fail within 150 hours (fig. S15). That means that the increased life span and lower overpotential in SACo@ZO-SE should be attributed to the functions of SE with atomic modulators instead of the PEO stabilizer.

### Interface analysis of cycled Li metal under low-temperature surroundings

**Fig. 4.**
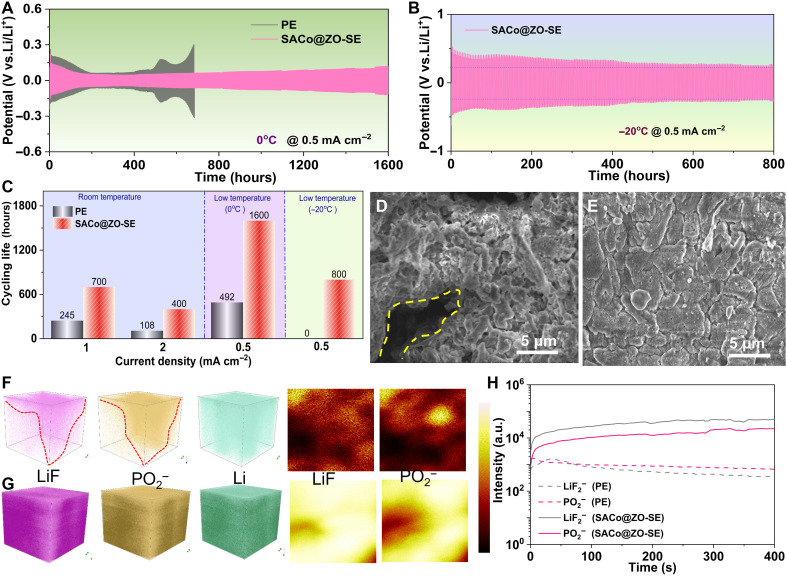
Interface analysis of cycled Li metal under low-temperature surroundings. The voltage curves of symmetric cell with PE or SACo@ZO-SE at 0.5 mA cm^−2^ under (**A**) 0°C and (**B**) −20°C, respectively. (**C**) The summary of the life span at various current densities under different temperature surroundings. The SEM images of cycled Li surface with the employment of (**D**) PE and (**E**) SACo@ZO-SE electrolyte under low temperature of 0°C. The three- and two-dimensional reconstruction of species distribution of Li metal based on (**F**) PE and (**G**) SACo@ZO-SE electrolyte. (**H**) The corresponding species distribution along with time increasing.

As known, the barriers of desolvation are more challenging under low temperatures, i.e., −20°C (*27*). Under 0°C, the cells with SACo@ZO-SE exhibit similar phenomena of long-term cycling, and they stabilize for 1600 and 800 hours under 0.5 and 1 mA cm^−2^ (Fig. 4A and fig. S16), respectively, which exceed the life span of the cells with PE. Even reducing the temperature to −20°C, the SACo@ZO-SE is capable of providing 800 hours for efficiently stripping and plating behaviors (Fig. 4B), while the cell with PE does not work owing to sluggish kinetics and severe barriers of Li^+^ solvation clusters. As summarized in Fig. 4C, the SACo@ZO-SE illustrates the temperature robustness without significantly sacrificing the life span even under the lower temperatures, indicating fast desolvation and diffusion under low temperature.

The postmortem analysis of Li plating morphology evolution was observed by the ex situ SEM (Fig. 4, D and E). Numerous random protrusions and pits cover on the cycled Li surface, and messy porous and dendritic Li is observed, exhibiting obvious crack. However, with the catalytic SACo@ZO in the SE, the morphology of Li deposition is completely changed from dendritic to spherical dense and uniform plating behaviors (*14*), indicating the stability of SACo@ZnO within the SE as well. As reconstructed by time-of-flight secondary ion mass spectrometry (TOF-SIMS) (Fig. 4, F to H), the obvious cracks and Li dendrites are formed in the PE, while the smooth plating behavior is achieved by the catalytic effect of SACo@ZO in the SE. Meanwhile, the surface species distribution intensities of LiF_2_^−^ and PO_2_^−^ interphases are also recorded (Fig. 4F), and they increase slightly (Fig. 4H), implying the weak interaction between solvents and central Li^+^, which are consistent with SEM results. These spectroscopic results have completely demonstrated that the SACo@ZO can affect the inner Helmholtz plane layer and speed up the desolvation kinetics at the interface to expedite Li^+^ diffusion. In addition, the stability of SEI and uniform plating revealed by TOF-SIMS and ex situ SEM confirms the high-efficiency in suppressing both dendritic protrusions and fracture development of SEI throughout repeated electrochemical plating/stripping.

### Revitalizing dendritic Li with atomic modulators

**Fig. 5.**
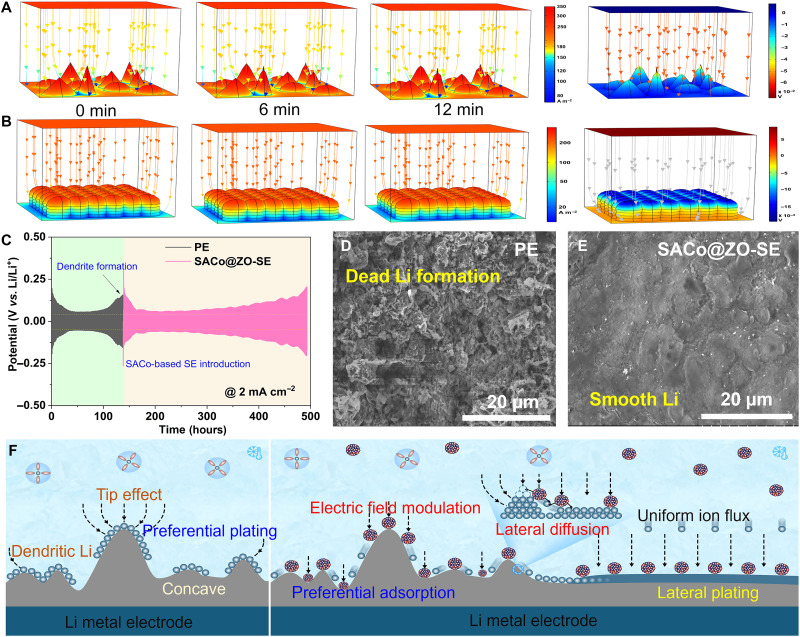
Revitalizing dendritic Li with atomic modulators. The COMSOL simulation of the Li plating behaviors and corresponding local electric field at 0, 6, and 12 min under (**A**) PE and (**B**) SACo@ZO-SE electrolyte. (**C**) The voltage curves before and after introducing SACo@ZO-SE electrolyte. (**D**) The SEM image of the dendritic Li formation under PE and (**E**) the dendritic Li evolution into smooth Li under the catalytic capability of SACo@ZO-SE. (**F**) Schematic illustration of SACo@ZO-based SE in self-expediting dendritic Li into lateral plating.

To imitate plating process, the finite element analysis is performed on COMSOL to visualize the functions of SACo@ZO in modulating local spatial distribution at the Li/electrolyte interface (*17*). In the PE system (Fig. 5A), because of the presence of random Li ion flux, the surface electric field exhibits a distinct gradual distribution, and the Li nucleation usually dominates at the tips, forming the excessive charge accumulation. However, with the adsorbed SACo@ZO (Fig. 5B), the electron-delocalized SACo@ZO alters the local strength of electric field, and the spatial concave surface is filled up with the catalytic nanoparticles, averaging the surface accumulation to exhibit the smooth plating morphology with uniform electric field.

To directly visualize this, the dendritic Li electrode was achieved after cycling in the PE (Fig. 5C), as confirmed by SEM with too many Li dendrites. A new cell used with dendritic Li, and fresh SACo@ZO-SE was assembled. At the beginning, the overpotentials start to decrease, and the cell stabilizes for another 300 hours. However, after refilling with fresh PE, the new cell somehow decreases the overpotential, but it keeps increasing after stabilizing for merely 30 hours, and last, it fails to remediate dendrites with the presence of the up-and-down voltage curves after cycling (fig. S17). As inspected by electrochemical impedance spectroscopy (EIS), it is indicated that the new cell does not experience an obvious short circuit after introducing fresh SACo@ZO-SE before and after cycling (Fig. 5, D and E, and fig. S18) (*34*, *53*). The dendritic or dead Li was revitalized by the catalytic SACo@ZO, and the dendritic morphology transforms into a uniform and dense deposition morphology with low lateral diffusion barrier. This demonstrates that SAC-based SE has the capability to drive the dendritic Li atom into a uniform and dense deposition (Fig. 5F), further evidencing that SACo@ZO promotes lateral diffusion of Li atom. Meanwhile, the interface adaptive electric field regulation of electron-delocalized SACo@ZO fundamentally regulates ion flux and charger distribution on the surface, allowing self-motivated behaviors of Li atom accelerated by SACo@ZO.

### Electrochemical performance of full cells under catalytic SE

**Fig. 6.**
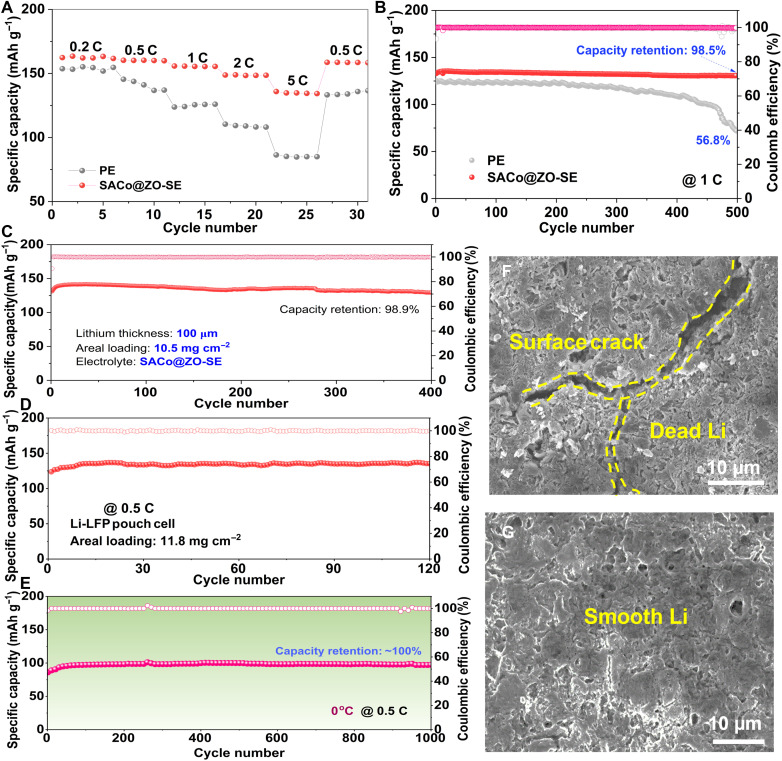
Electrochemical performance of full cells with revitalized Li metals. The comparison of (**A**) rate performance and (**B**) cycling performance at 1 C of the Li-LFP full cell based on PE or SACo@ZO-SE electrolyte. (**C**) The electrochemical stability evaluation of the high-loading Li-LFP full cell with SACo@ZO-SE electrolyte under thin Li foil. (**D**) The cycling performance of the high areal pouch cell based on SACo@ZO-SE electrolyte. (**E**) The low-temperature performance of the full cell based on SACo@ZO-SE electrolyte under 0°C. The surface morphology of the cycled Li with (**F**) PE and (**G**) SACo@ZO-SE electrolyte.

A series of Li-LiFePO_4_ (Li-LFP) full cells used with PE or SACo@ZO-SE is assessed (1C = 170 mA g^−1^). The full cell used with SACo@ZO-SE delivers rate capacities of 162, 156, and 135 mA hour g^−1^ at 0.2, 1, and 5 C (Fig. 6A), respectively, which overwhelms the PE one (85 mAh g^−1^ at 5 C). Meanwhile, the cell with SACo@ZO-SE only displays the polarization voltage of 160 mV at 5 C (fig. S19), indicating the faster Li kinetics. Cycled at 1 C (Fig. 6B), an initial capacity of 133 mA hour g^−1^ and extremely high capacity retention of 98.5% after 500 cycles are obtained. For practical applications, the low negative/positive (N/P) ratio and high mass loading cathode for batteries are evaluated under low temperature. With thin Li foil (50 μm), the full cell stabilizes the capacity of 139 mA hour g^−1^ after 250 cycles at 1 C (fig. S20). Simultaneously enhancing the mass loading to 10.5 mg cm^−2^, it still showcases the final capacity of 129 mA hour g^−1^ with the capacity retention of 98.5% for 400 cycles (Fig. 6C). Enlarging to a pouch cell, it remains to stabilize for 120 cycles with a capacity retention of ~100% at 0.5 C (Fig. 6D and fig. S21).

Under 0°C, the cell with SACo@ZO-SE shows the long-term cyclability of 1000 cycles and remains the capacity retention of ~100% at 0.5 C after 1000 cycles (Fig. 6E) thanks to the self-revitalizing behaviors of SACo@ZO in delocalizing dendritic Li atoms with the uniform spatial electric field and accelerated Li-ion flux. To further demonstrate this point, the anode-free cells of Cu-LiNi_0.8_Co_0.1_Mn_0.1_O_2_ (NCM811) used with PE or SACo@ZO-SE were assembled and evaluated (fig. S22). Under the loading of 10 mg cm^−2^, it is found that the Cu-NCM811 cell with SACo@ZO-SE delivers higher capacity and Coulombic efficiency than that with PE at 0.2 C (1 C = 180 mA g^−1^), further verifying the fact of delocalizing the Li plating on pristine Cu by catalysis of the SACo@ZO. This also indicates that the morphology of SACo@ZO is relatively stable throughout electrochemical plating/stripping processes, thereby enhancing Li^+^ desolvation kinetics and uniformizing Li electrodeposition (*54*). Afterward, the surface morphology of cycled Li electrode was also investigated by SEM (Fig. 6, F and G). In comparison with Li anode in the PE with obvious cracks and dendrites, the SACo@ZO-SE modulated Li electrode has a smooth and dense morphology, highlighting the catalytic effect in revitalizing dendritic Li for lateral deposition.

## DISCUSSION

In summary, the strategy of SE using electron-delocalized atomic modulators in commercial PE is proposed to revitalize dendritic Li for long-term life span. The atomic metal anchored on metal oxides as the catalytic sites has initially dispersed, regulating spatial electric field at the interface and weakening the interactions between Li^+^ and solvents. The desolvation evolutions in Helmholtz plane layer have been monitored by in situ SFG spectroscopy under various bias voltages. The catalytic SACo@ZO-assisted suspension electrolyte enables the dynamic rehabilitation effect toward dendritic Li, as confirmed by simulations and electronic microscopy. As a result, a smooth but dense Li plating behavior is achieved under room or low-temperature surroundings. The optimized high areal loading LiFePO_4_ pouch cell with a SACo-decorated SE lasts for 1000 cycles and keeps the capacity retention of ~100% under 0°C, demonstrating the feasibility of atomic catalytic SE against dendritic Li for practical application.

## MATERIALS AND METHODS

### Synthesis of SACo@ZO catalyst

Initially, the commercial powder of zinc oxide (ZnO) was reduced under 300°C for 2 hours with the heating rate of 5°C min^−1^ under H_2_/Ar (5%:95%, by volume), and the defect-rich zinc oxide was obtained (ZnO_1−*x*_) after cooling down to room temperature. Then, the above-pretreated powder (244.14 mg) and cobalt nitrate hexahydrate (17.46 mg) were added into anhydrous ethanol (20 ml) and kept stirring for 24 hours before evaporating ethanol under 90°C. Last, the obtained solid powder was treated under 200°C for 1 hour with the heating rate of 5°C min^−1^ under the atmosphere of NH_3_/Ar (10%:90%, by volume), generating the electron-delocalized single atomic cobalt on defect-rich ZnO_1−*x*_ (denoted as SACo@ZO) composite.

### Synthesis of SACo@ZO-SE

The commercial electrolyte containing 1 M LiPF_6_ dissolved in a mixed solvents of ethylene carbonate/dimethyl carbonate/ethylmethyl carbonate (EC/DMC/EMC) (v:v:v = 1:1:1) with 10 wt % fluoroethylene carbonate (FEC) is adopted as the PE. In the Ar-filled glove box, the 50 mg of SACo@ZO is added into 10 ml of PE and kept stirring with the weight percentage of 2 wt % of the polyethylene oxide stabilizer (PEO, molecular weight (M_w_) = 600,000) for overnight, forming the SACo@ZO-SE electrolyte. Before assembling cells, the as-prepared SACo@ZO-SE is stirred for 10 min to completely generate homogenous dispersion.

### Cell fabrication

All CR2025-typed coin cells were assembled in the glove box filled under the atmosphere of high-pure argon and less moisture/O_2_ (<0.1 parts per million). Without any specific information, the thickness of the commercial Li foil is 450 μm, and the diameter is 15.6 mm. For the cells, 60 μl amount of SACo@ZO-SE or PE was added into the symmetrical cells or asymmetrical cells, where the excessive electrolyte will flow out of coin cells. The commercial LiFePO_4_ electrode with the mass loading of 3 or ~12 mg cm^−2^ is adopted for the full cell measurements. The commercial NCM811 electrodes with the mass loading of ~10 mg cm^−2^ are adopted for assembling anode-free Cu-NCM811 cells.

### Characterization method

The field-emission scanning electron microscope (Quanta FEG 250) is selected to observe the morphology of SACo@ZO and the cycled Li surface morphology with or without SACo@ZO-SE. Raman spectroscopy (LabRam, HR, Evolution) with 532-nm laser wavelength is used to investigate the solvation structures in PE or SACo@ZO-SE. The crystal structures of the synthesized materials with/without SACo were visualized by XRD (Bruker D8). The atomic presence of Co was investigated via XAS experiments at the Shanghai and Beijing Synchrotron Radiation Facility and the aberration-corrected scanning transmission electron microscope at 300 kV. The surface morphology and species distribution of cycled Li electrode were measured by TOF-SIMS. Electrochemical impedance spectroscopy (EIS) measurements of the coin cells were completed on a VMP-3 electrochemical working station with the frequency ranging from 200 kilohertz to 100 millihertz. The Land CT2001 automatic battery tester was adopted, and the areal plating/stripping capacity was fixed at 1 mA hour cm^−2^ at designate current densities. For the Coulombic efficiency measurement, the stripping cutoff voltage is set up to 1.0 V (versus Li/Li^+^). To investigate the elemental chemical environments and valence states of the synthesized materials, the x-ray photoelectron spectroscopy (XPS) was performed on a Thermo Fisher Scientific spectrometer (ESCALAB250XI). The in situ/operando SFG was performed on commercial device under various bias potentials. The visible light wavelength was set at 532 nm, and the infrared pulse ranges from 2700 to 3000 cm^−1^, where the lights were directly shone on the electrode/electrolyte interface.

### COMSOL Multiphysics simulation

The COMSOL Multiphysics 6.1 was adopted to simulate the current density distributions and lithium ion concentration on the metallic Li surface based on the three-dimensional transient model and tertiary Nernst-Planck Eq. 1, where the electron number and initial Li^+^ concentration are set as 1 and 1 M, respectively. According to Fick’s law and Poisson’s equation, the Li^+^ diffusion was affected by the concentration gradient and the distribution of electric field. During electrochemical operation, the electrode system follows the principle of electronic charge conservation and mass conservation

$$J_{i}\left(x\right)=-D_{i}\frac{\partial C_{i}\left(x\right)}{\partial x}-\frac{z_{\text{iF}}}{\text{RT}}D_{i}C_{i}\frac{\partial \Phi \left(x\right)}{\partial x}+C_{i}v\left(x\right)$$ (1)

where *J_i_*(*x*) is the flow rate of substance *i* at the distance *x* from the electrode surface (in moles per second per square centimeter); *D_i_* is the diffusion coefficient (in square centimeters per second); $\frac{\partial C_{i}\left(x\right)}{\partial x}\;$ is the concentration gradient at *x*; $\frac{\partial \Phi \left(x\right)}{\partial x}\;$ is the potential gradient; *Z_i_* and *C_i_* are the charge and concentration of substance *i*, respectively, and the three items on the right of the formula represent the contribution of diffusion, migration and convection to the flow.

### Simulation method

The calculations are based on density functional theory (DFT) and molecular dynamics simulations as implemented in the Quantum ATK. The nonspin polarization and spin-polarized DFT are used for pristine ZnO and SACo@ZO systems, respectively. The exchange-correlation potential is explained by the Perdew-Burke-Ernzerhof functional based on spin-generalized gradient approximation plus on-site Coulombic interaction (SGGA + *U*) functional with *U*_eff_ = 3 eV on Co *3d*. The projector augmented-wave method is used for wave function expansion with an energy cutoff of 450 eV. The geometry optimization continues until the energy differences and ionic forces converge to less than 10^−6^ eV and 0.01 eV Å^−1^, respectively. The Monkhorst-Pack *k*-point meshes of 13 × 13 × 1 are used for electronic structure calculation of metal oxides (including Co atom and Li ion adsorption). Calculation of desolvation energy barriers of Li(EC)_4_^+^ into Li(EC)_3_^+^ and EC on the different electron-delocalized catalyzers is set *k*-point meshes of 5 × 5 × 1. For the slab calculations, the vacuum thickness was chosen to be 15 Å to reduce artificial interactions due to periodic boundary conditions.

The RDF allows the interaction of lithium ions with other components of the system and is calculated as follows

$$g_{\mathrm{ij}}=\frac{V}{N_{i}N_{j}}\sum_{i=1}^{N_{\mathrm{ij}}}\frac{n_{\mathrm{ij}}}{4\pi r^{2}∆r}$$

where *V* is the volume of the box, *N_i_* and *N_j_* are the numbers of *i* and *j* particles, and *n_ij_* is the number of ions in the spherical shell between radius *r* and radius *r* + Δ*r* with the *j* particle.
