# Supplementary material for: Revitalizing dendritic lithium with atomic modulator-decorated suspension electrolyte for durable lithium metal batteries
Source: Sci Adv. 2026 May 22;12(21):eaef9111. doi: 10.1126/sciadv.aef9111 (PMC13196776; doi:10.1126/sciadv.aef9111)
Supplement: Supplementary file 1 — Figs. S1 to S22 [file sciadv.aef9111_sm.pdf]

Science Advances
AAAS

Supplementary Materials for

Revitalizing dendritic lithium with atomic modulator-decorated suspension electrolyte for durable lithium metal batteries

Jian Wang *et al.*

Corresponding author: Jian Wang, jian.wang@kit.edu; Jing Zhang, zhangjing2020@xaut.edu.cn; Hongzhen Lin, hzlin2010@sinano.ac.cn; Maximilian Fichtner, maximilian.fichtner@kit.edu

Sci. Adv. **12**, eaef9111 (2026)
DOI: 10.1126/sciadv.aef9111

This PDF file includes:

Figs. S1 to S22

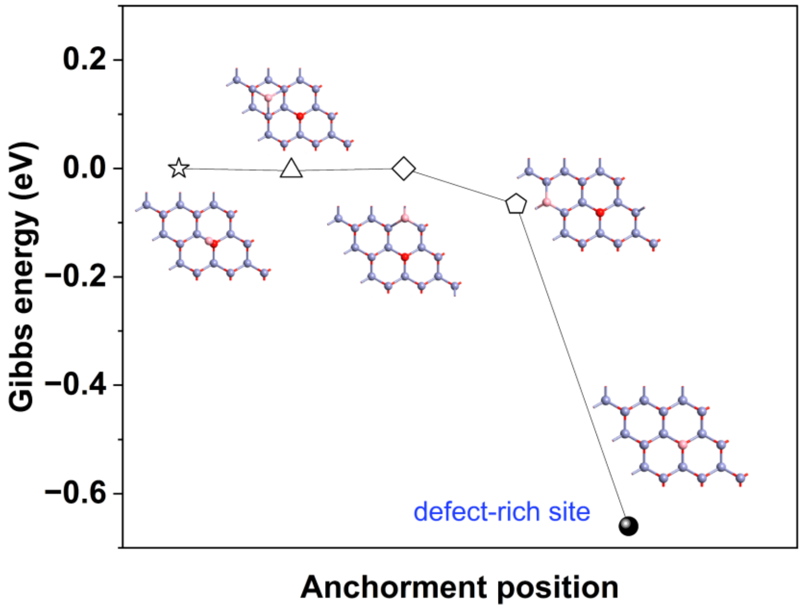

Fig. S1. Simulation on SAC-in-defect structure. Comparison of Gibbs energy by moving Co atom on different sites of defect-rich ZnO.

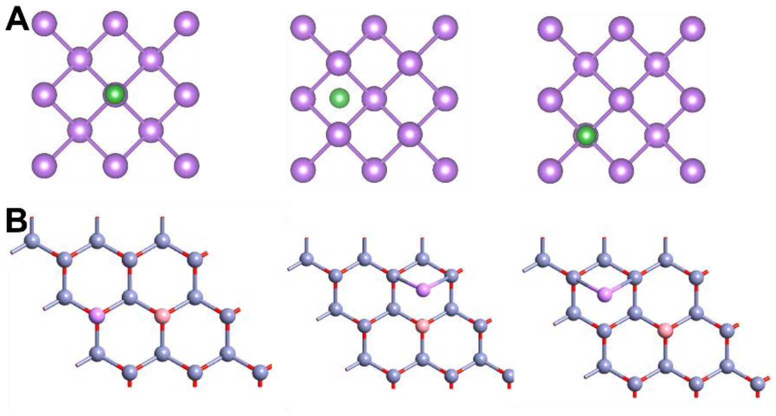

Fig. S2. Simulated structures of Li atom on matrix. The simulated images of Li atom diffusion on (A) metallic Li and (B) SACo@ZO.

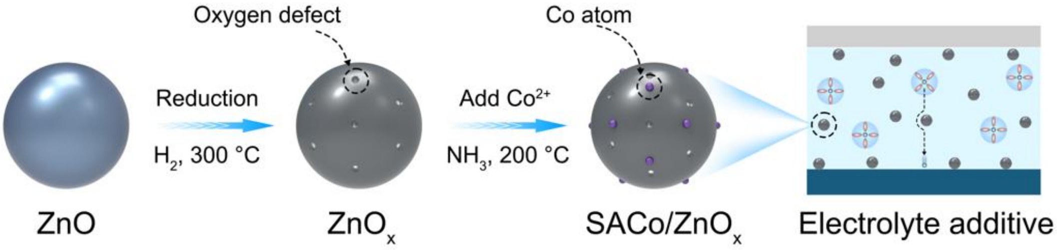

Fig. S3. Synthesis process of SACo@ZO. The fabrication process of SACo@ZO, serving as the suspension electrolyte additive.

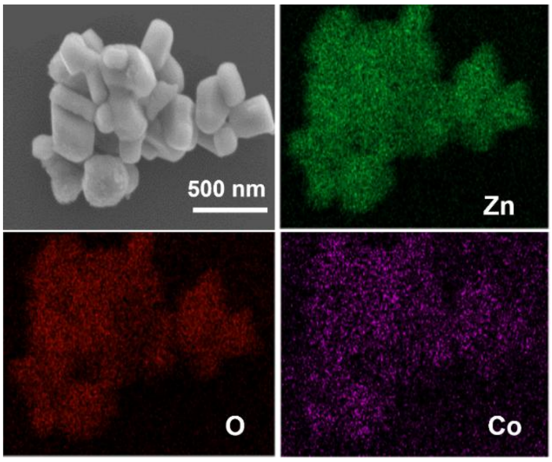

Fig. S4. SEM mappings of SACo@ZO. The morphology and corresponding elemental distribution of SACo@ZO nanoparticle.

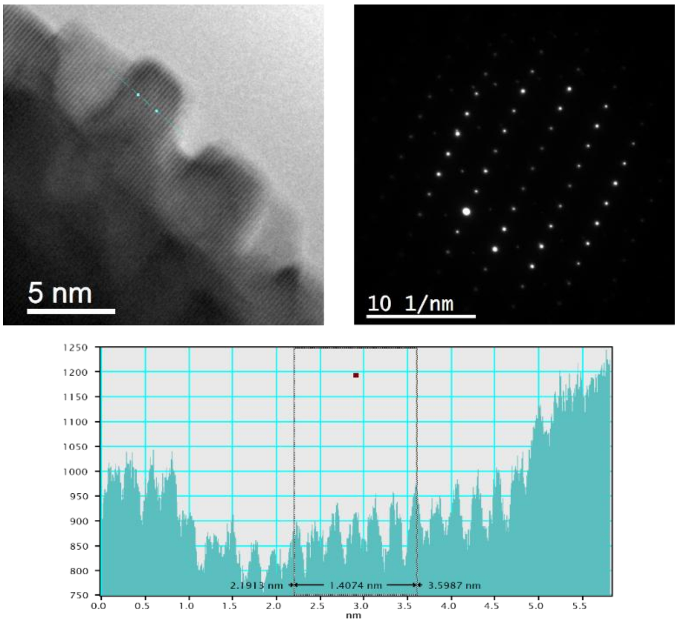

Fig. S5. TEM tests of SACo@ZO. The high-resolution TEM image and corresponding layer spacing of SACo@ZO.

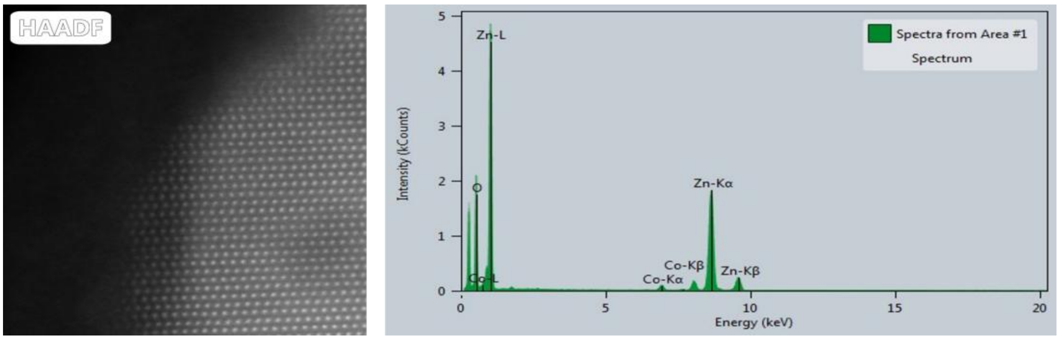

Fig. S6. Atom-level investigation of SACo@ZO. The HAADF image and corresponding Co presence of SACo@ZO catalyst.

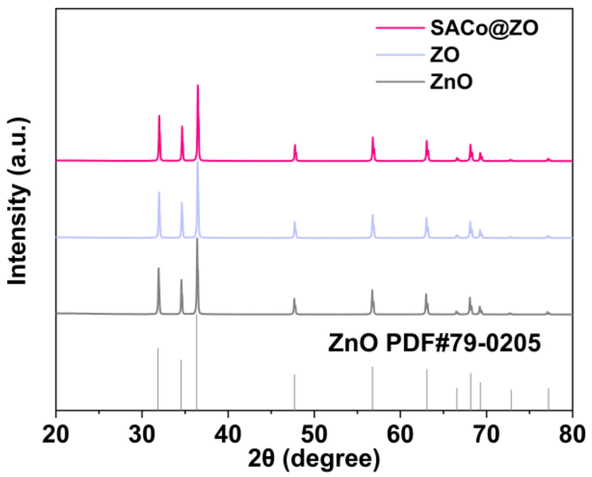

Fig. S7. XRD measurements. The XRD patterns of these composites.

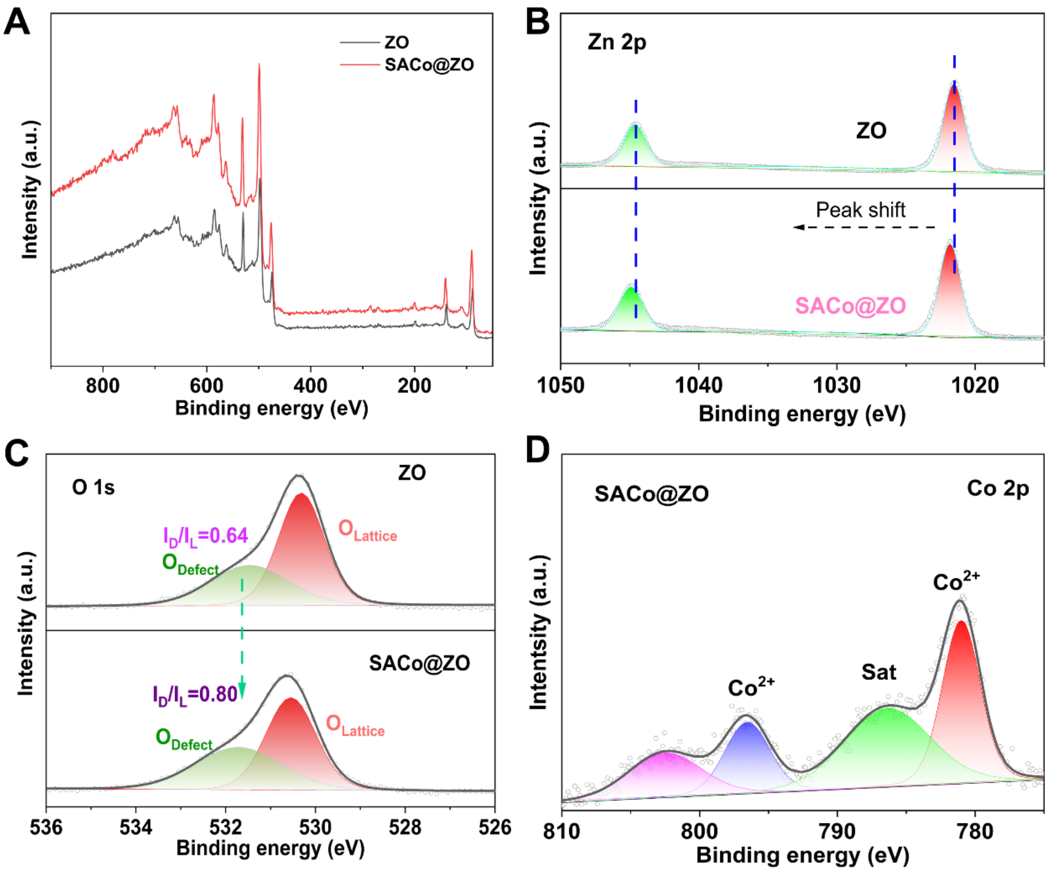

Fig. S8. XPS measurements of two composites. The comparison of (A) wide spectrum; the high-resolution XPS of (B) Zn 2p and (C) O 1s of the two samples with/without SACo. (D) The high-resolution Co XPS spectrum of SACo@ZO.

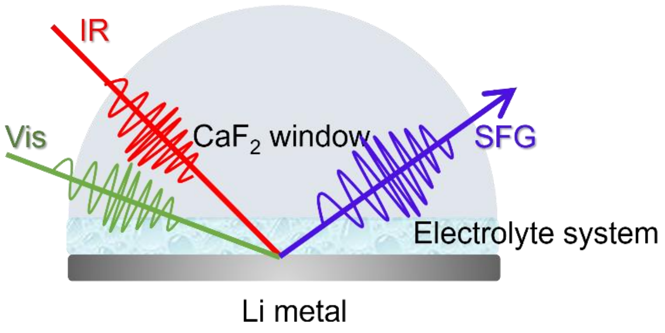

Fig. S9. Schematic illustration of in-situ SFG cell. The illustration of in-situ/operando SFG cell on the interface evolution measurement.

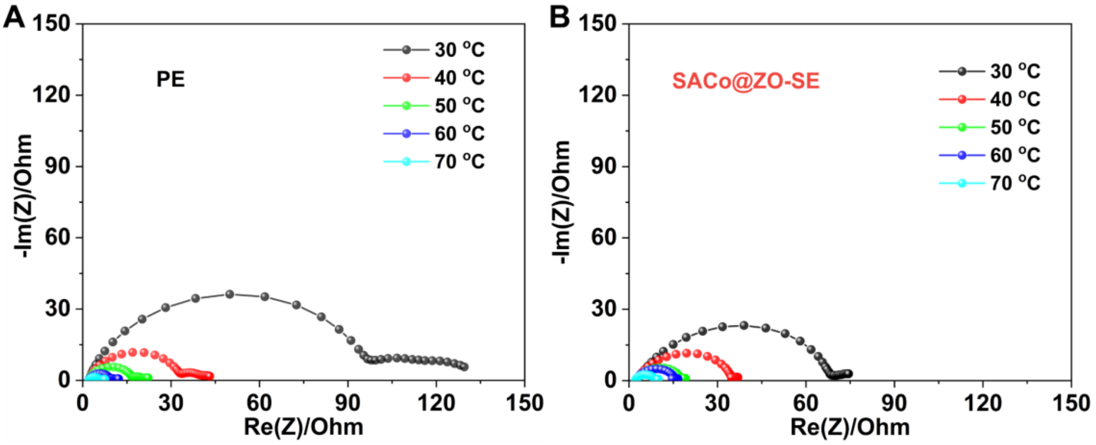

Fig. S10. Temperature-dependent EIS of Li-Li cells. The EIS evolution of Li symmetric cells based on (A) PE and (B) SACo@ZO-SE electrolyte under different temperatures.

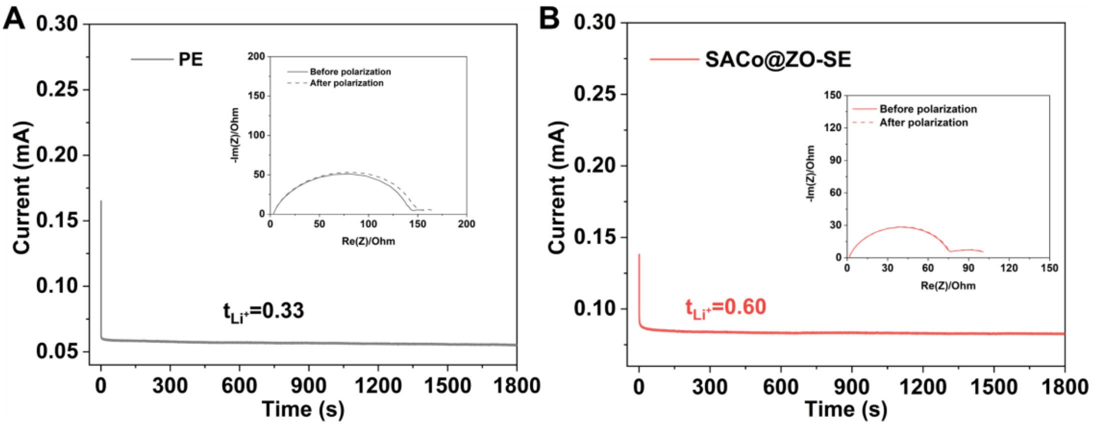

Fig. S11. Li$^+$ transference number. The Li$^+$ transference number of symmetric cells based on (A) PE and (B) SACo@ZO-SE electrolyte.

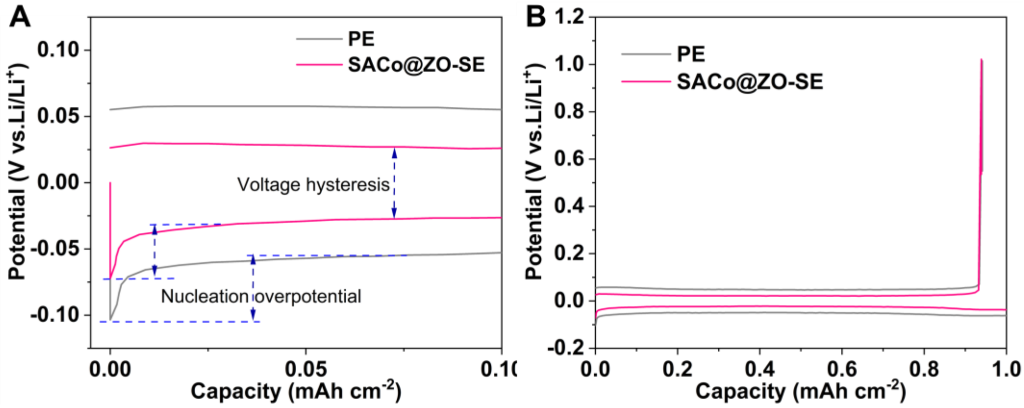

Fig. S12. Nucleation overpotential and voltage hysteresis. (A) The nucleation overpotential and (B) corresponding voltage curves of Li-Cu with PE or SACo@ZO-SE electrolyte at the first stripping/plating process, respectively.

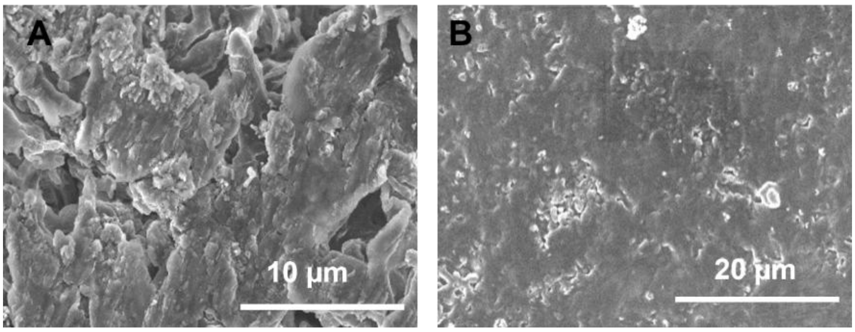

Fig. S13. SEM images of Cu with Li pre-plating. The SEM image of plating 1 mAh cm^{-2} Li on Cu based on (A) PE and (B) SACo@ZO-SE electrolyte.

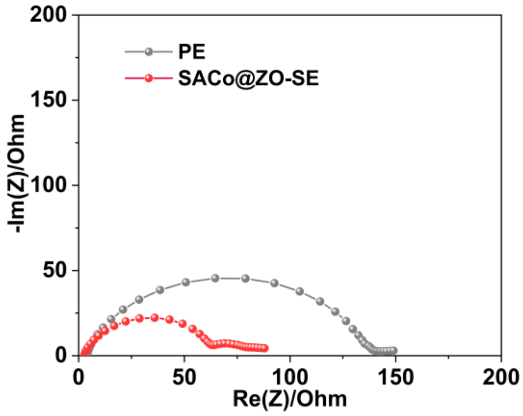

Fig. S14. EIS of symmetric Li-Li cell. The comparison of symmetric Li-Li cell based on PE or SACo@ZO-SE electrolyte.

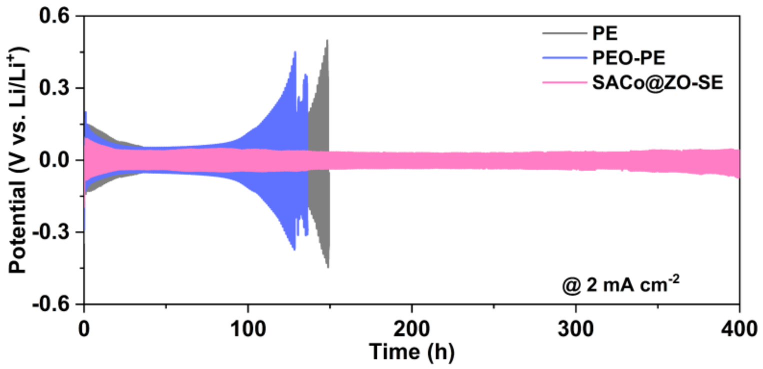

Fig. S15. Voltage curves of Li symmetric cell. The voltage curves of the Li symmetric cells employed with PE, PEO-PE or SACo@ZO-SE at 2 mA cm^{-2}.

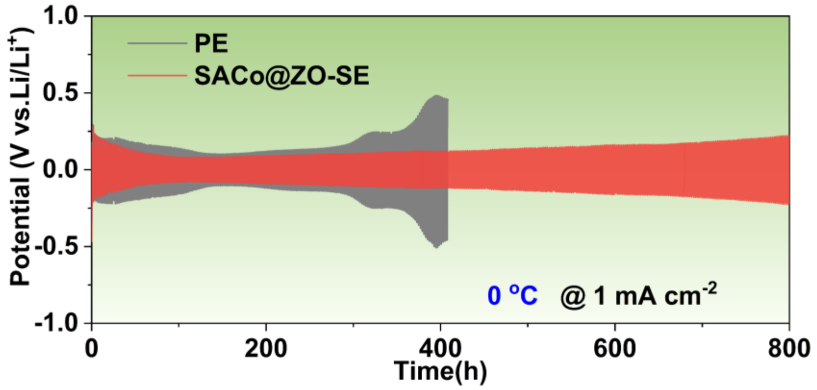

Fig. S16. Voltage curves of Li symetric cell under low temperature. The voltage curves of the symmetric cells employed with PE or SACo@ZO-SE at 1 mA cm^{-2} under 0 ºC.

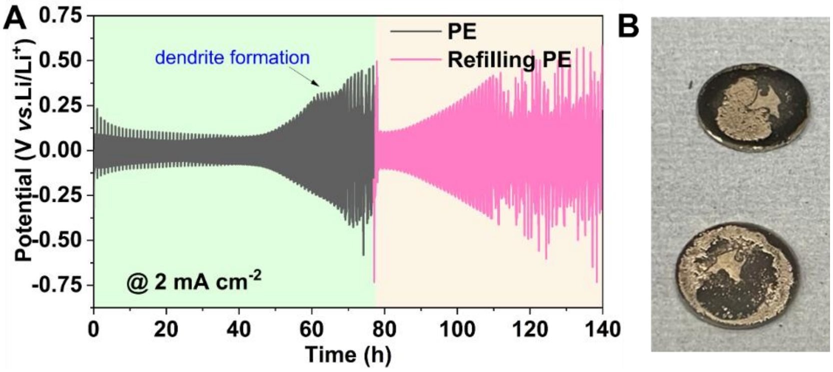

Fig. S17. Voltage curves and optical images of dendritic Li before and after refilling PE. (A) The voltage curves of dendritic Li electrode after refilling with fresh PE; (B) The optical images of the dendritic Li electrodes.

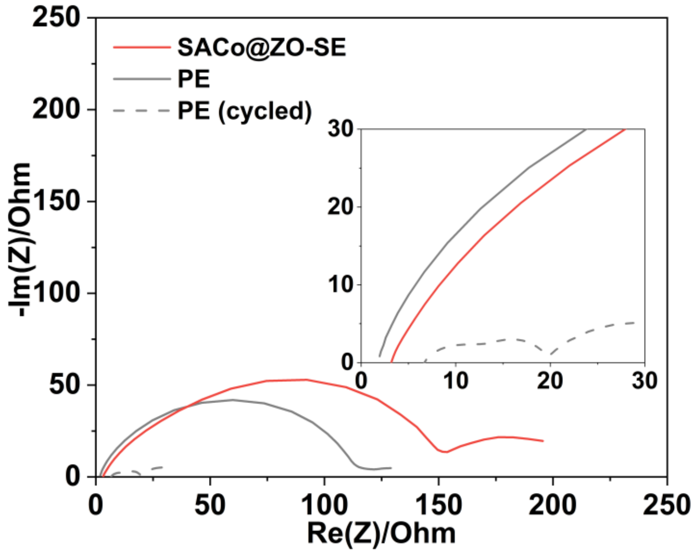

Fig. S18. EIS of dendritic Li-Li cell before and after revitalization. The EIS evolution of the "dendritic Li" cell with PE or introducing SACo@ZO-SE electrolyte.

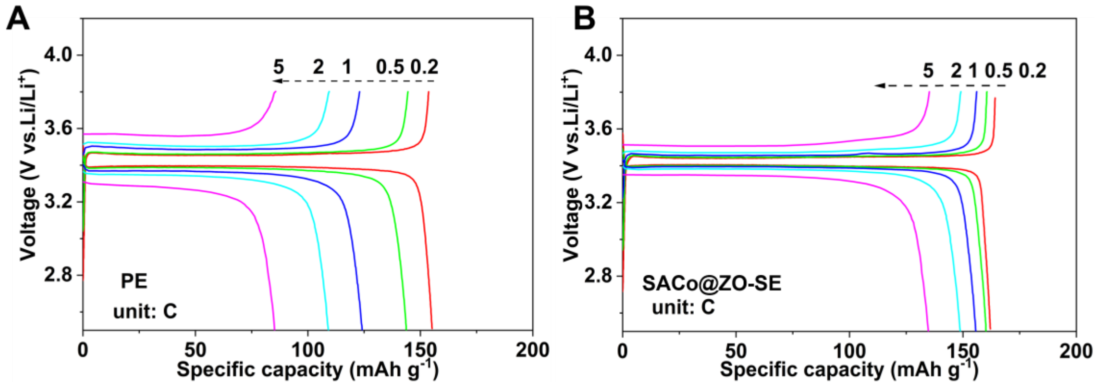

Fig. S19. Voltage behaviors of Li-LFP full cells. The voltage curves of the Li-LFP full cells employed with (A) PE and (B) SACo@ZO-SE under different current rates, respectively.

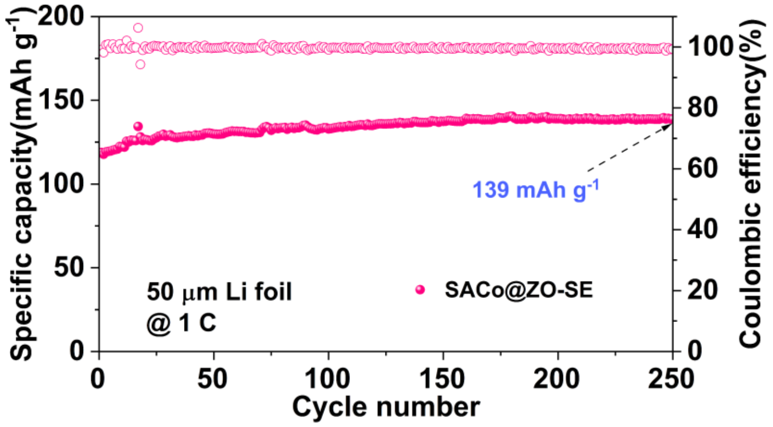

Fig. S20. Cycling stability of Li-LFP with 50 μm Li foil. The cycling performance of the Li-LFP full cell employed with SACo@ZO-SE electrolyte with thinner Li foil under 1C.

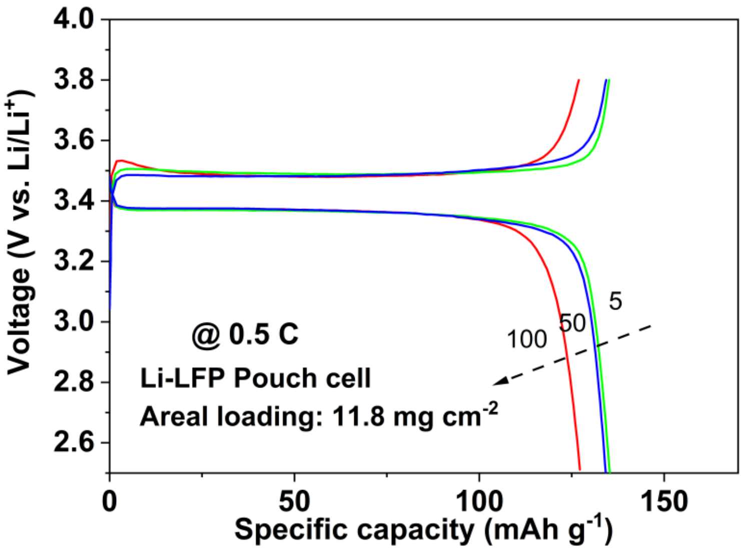

Fig. S21. Voltage behaviors of pouch cell. The voltage curves of the Li-LFP pouch cell with SACo@ZO-SE under different cycles.

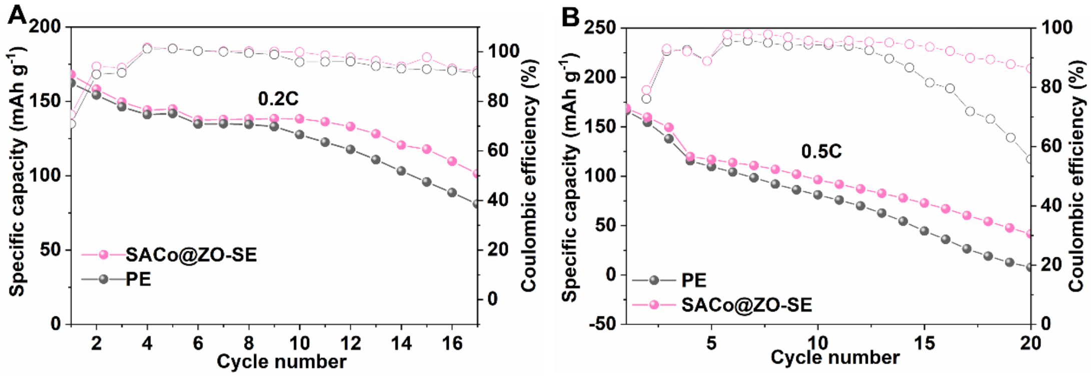

Fig. S22. Cycling performance of Cu-NCM811 full cell. Cycling performance of the (A) anode-less (1 mAh cm^{-2} Li amount on Cu) and (B) anode-free Cu-NCM811 cells employed with PE or SACo@ZO-SE.
